# Reduction and Alkylation of Proteins in 2D Gel Electrophoresis: Before or after Isoelectric Focusing?

**DOI:** 10.3389/fchem.2017.00059

**Published:** 2017-08-15

**Authors:** Xiaolin Wu, Chenhui Xu, Wei Wang

**Affiliations:** State Key Laboratory of Wheat and Maize Crop Science, Collaborative Innovation Center of Henan Grain Crops, College of Life Sciences, Henan Agricultural University Zhengzhou, China

**Keywords:** 2D gel, proteomics, sample preparation, isoelectric focusing, SDS-PAGE, strip equilibration

## Introduction

Two-dimensional gel electrophoresis (2DE) is a well-developed, high-resolution technology to separate complex mixtures of proteins. This technique has been widely used for the past four decades, and has played a vital part in the developmental history of proteomics. The advantages and limitations of 2DE in proteomics research have been reviewed (e.g., Thelen and Peck, 2007; Oliveira et al., 2014; Ning et al., 2016; Padula et al., 2017). Currently, gel-free proteomics is the most widely used proteomics workflows; however, 2DE still has a niche in proteomics and beyond (Rabilloud, 2012; Rogowska-Wrzesinska et al., 2013; Oliveira et al., 2014; Arentz et al., 2015; Ning et al., 2016; Padula et al., 2017), as 2DE and its derivative technologies [e.g., difference gel electrophoresis (DIGE)] are highly suitable for separation of intact proteoforms (protein isoforms) and analysis of protein post-translational modifications (Tannu and Hemby, 2006; Arentz et al., 2015; Padula et al., 2017).

**Figure 1:**
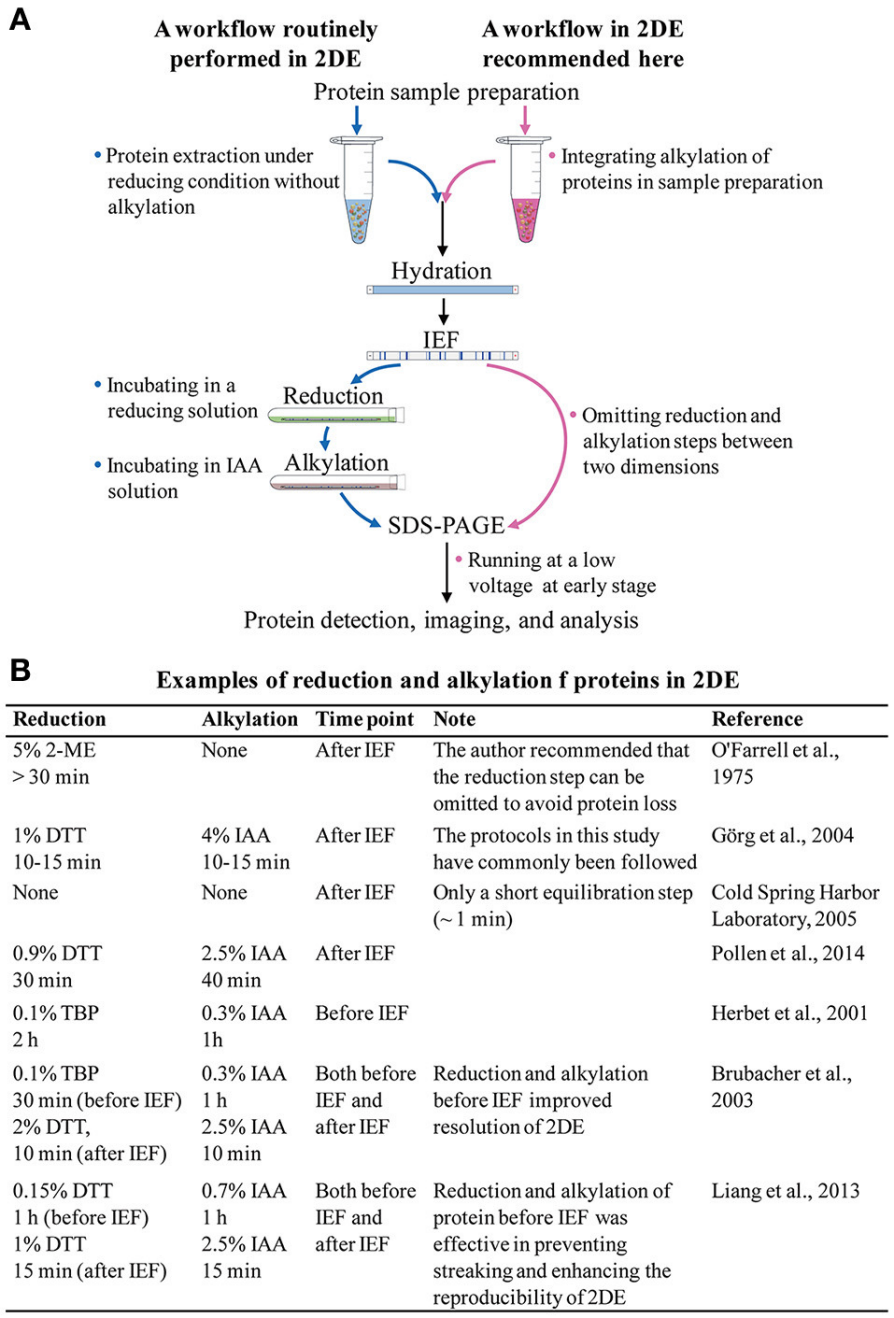
**(A)** The routinely used 2DE workflow, and a suggested 2DE workflow. **(B)** Examples of reduction and alkylation of proteins in 2DE.

In theory, the current 2DE technique is the same as the original method developed by O'Farrell (1975), i.e., the proteins are separated first by their isoelectric points in the first dimension of isoelectric focusing (IEF), and then by their molecular masses in the second dimension of sodium dodecyl sulfate polyacrylamide gel electrophoresis (SDS-PAGE). In practice, however, 2DE consists of several major steps, including protein extraction, IEF, after-IEF equilibration, SDS-PAGE, and protein visualization (Figure 1A).

The after-IEF equilibration step is required for the reduction and alkylation of focused proteins. Since 2DE is a well-established technology, researchers working with 2DE have routinely carried out the after-IEF equilibration step without any proper understanding of its necessity. Therefore, it is time for us to rethink and optimize our approach to after-IEF equilibration in 2DE. In this article, we review the origin, protocol, and effects of after-IEF equilibration in 2DE. We also suggest incorporation of the reduction and alkylation of proteins into the sample preparation protocol before 2DE, and the omission of the after-IEF equilibration step.

## Chemical reactions in after-IEF equilibration

The major rationale for after-IEE equilibration is that it prevents point streaking and other silver-staining artifacts, which are associated with excess dithiothreitol (DTT) in the protein sample (Görg et al., 1987). After-IEF equilibration is always carried out by incubating focused gel strips in an SDS-containing buffer for an appropriate time (Figure 1B). The initial equilibration included only a single reduction step (>30 min) in an SDS sample buffer plus 5% (v/v) 2-mercaptoethanol (2-ME; O'Farrell, 1975). With the introduction of immobilized pH gradient (IPG) strips, a two-step protocol was developed by Görg et al. (1987, 1988): first, reduction of proteins with a thiol-reducing reagent (commonly using DTT); and, second, alkylation of proteins with iodoacetamide (IAA). The IPG strips make 2DE much easier and more reproducible, and the after-IEF equilibration step recommended in the guidelines for IPG strips (Görg et al., 1988) is used widely in laboratories.

During IEF, thiol-reducing reagents such as, DTT are used for reduction of disulfide bonds and complete unfolding of proteins. However, DTT becomes negatively charged during IEF and migrates away from the basic end of the IPG strip toward the anode, which allows intramolecular and intermolecular disulfide bonds to reform. As a result, minor artifacts, including poorly focused and blurred spots, comet-like spot streaks, and the disappearance or appearance of some spots, can be formed (Herbert et al., 2001). As a remedy, excess amounts of DTT or 2-ME can be used in the first step of after-IEF equilibration for reduction of the reformed disulfide bonds during IEF. Thereafter, IAA can be used in the second step to alkylate the free thiol groups of cysteine residues and prevent their reoxidation during SDS-PAGE, as well as remove excess amounts of DTT (Görg et al., 1987). By excluding DTT and 2-ME from the IEF gel equilibration buffer, point streaking can be eliminated; however, blurred protein spots in the 2D map of leukemia proteins were observed (Görg et al., 1987). Moreover, urea and glycerol in the equilibration buffer have been shown to considerably improve protein transfer, whereas thiourea is not recommended because it causes streaks in 2DE patterns (Görg et al., 2004) by neutralizing IAA (Herbert et al., 2001). In summary, DTT may improve 2DE maps to an extent, but if used in excess it produces additional, potentially adverse effects on 2DE patterns (Görg et al., 1987). We carefully evaluated the work of Görg et al. (1987) and concluded that after-IEF equilibration did not significantly improve the quality of 2DE maps.

## What needs to change in 2DE workflow

The rationale for after-IEF equilibration has been questioned before (Zhou et al., 2005; Van et al., 2014), but its critics have not received enough attention. For good resolution of proteins by IEF, a requirement is that proteins are completely reduced and quantitatively dissolved. A previous study demonstrated that the after-IEF approach cannot really solve the problem of -SH re-oxidation of proteins. Instead, reduction and alkylation prior to any electrophoretic step, including IEF, seems to be the optimal approach to generate artifact-free 2DE maps (Herbert et al., 2001). A recent study also showed that reduction and alkylation prior to IEF were more effective in preventing the horizontal streaking caused by -SH oxidation (Liang et al., 2014).

In fact, -SH oxidation of proteins occurs in any electrophoretic process. Therefore, the reduction and alkylation of proteins after IEF can only prevent -SH oxidation that occur during SDS-PAGE, and it is of no help with the problem of -SH re-oxidation occurring during IEF, which may lead to horizontal and vertical tailing in 2DE gels. Therefore, in order to completely eliminate the re-oxidation of -SH groups, reduction and alkylation should be carried out before IEF. To date, however, only a few studies have suggested this ordering of steps (Herbert et al., 2001; Brubacher et al., 2003; Liang et al., 2014).

Another rationale for after-IEF equilibration is that it helps to promote protein transfer from IEF strips to SDS gels. It has been suggested that the focused proteins bind strongly to the IPG gel matrix (with fixed changed groups), and that after-IEF equilibration coats all proteins with SDS, thereby improving protein migration in SDS-PAGE (Rabilloud and Lelong, 2011). As a result, a prolonged equilibration was often carried out (e.g., 30 min reduction +40 min alkylation, Pallen et al., 2014).

However, with this approach, focused proteins, especially those located on the surfaces of the IPG strips, may be lost. O'Farrell (1975) found a 5–25% loss of focused proteins, depending on the protein sample and equilibration duration. Later studies reported up to 20% protein loss (Görg et al., 2004; Zhou et al., 2005), resulting in variability in 2DE patterns. Clearly, then, after-IEF equilibration increases the gel-to-gel and run-to-run variability of comparative proteomics analysis, which depends on equal protein load among several samples. This may explain the inconsistency in protein loads and observed protein amounts (lower) that sometimes occurs in 2DE. Therefore, promotion of protein transfer by after-IEF equilibration is partly offset by a wash-off effect. In addition, prolonged after-IEF equilibration causes the spots to swell and become less sharp.

## Performing protein reduction and alkylation before IEF

To overcome the problem of protein loss, the classic protocol by Cold Spring Harbor Laboratory (in association with *Nature Methods*, Cold Spring Harbor Laboratory, 2005) recommends that, after IEF, the gel strip is briefly equilibrated in the SDS-containing buffer and placed directly onto the top edge of a second-dimension lab gel. As a result, the total time between the end of IEF and the beginning of SDS-PAGE should ideally be just a few minutes, thereby minimizing diffusion. With this protocol, the protein spots are highly resolved and nearly round in shape. However, this protocol has not been widely applied, probably because it requires laboratory-cast IEF gel rather than commercial IPG strips.

In fact, in the presence of SDS, the migration of proteins from IPG strips to the SDS gel in the electric field is never a problem. The widely used running buffer system (Laemmli, 1970) contains 0.1% SDS. In our laboratory, the after-IEF equilibration is omitted (Figure 1A). Instead, we place 0.2–1.0 ml (depending on the dimensions of the gel slabs) of 1% SDS sample buffer (pH 8.8) in the strip well and place the IPG strip in it. Then, we start the SDS electrophoresis at a low voltage so as to allow time for the SDS front to re-solubilize the proteins while sweeping across the IPG strip. This protocol greatly facilitates protein transfer and allows us to obtain good 2DE maps. No apparent difference was observed between the gels with and without after-IEF equilibration.

Reduction and alkylation are essential for proper 2DE map analysis. However, if proteins have been reduced before IEF, why do they need to be reduced again afterward? Even if individual proteins in a complicated sample are not completely reduced prior to IEF, attempting to remedy this by after-IEF equilibration is not ideal, as vertical or horizontal tailing (smearing) in the 2DE patterns cannot be avoided owing to the change in molecular masses of reduced protein spots. Optimizing the sample preparation protocol to contain a reduction and alkylation step is a better option. For example, the Bio-Rad Laboratory showed that reduction alkylation treatment during sample preparation led to improved 2DE maps, significantly reducing horizontal streaking (Brubacher et al., 2003). Previous studies have shown that no apparent alterations in protein patterns are observed during SDS-PAGE in the presence of IAA (Görg et al., 1987; Herbert et al., 2001). However, protocols for pre-reduction and alkylation may vary greatly depending on experimental conditions and materials, and need to be optimized. For example, the optimal concentration, treatment time, duration, and potential effectiveness of reducing agents in IEF should be characterized.

At present, a challenge for the routine application of 2DE is to standardize the technique and ensure its reproducibility. The availability of IPG strips and pre-cast SDS gels such as, those produced by Criterion means that 2DE can be effectively semi-automatic. The preparation of reducing and alkylation agents, and the two steps of after-IEF equilibration involved, are cumbersome and time-consuming, and not readily compatible with the automatic design and operation of 2DE. Currently, the majority of laboratories worldwide are using after-IEF equilibration, thereby wasting time. If the reduction/alkylation step can be completed before IEF and incorporated into sample preparation, this problem can be easily resolved, 2DE will run fast (a process being reduced by at least 30 min), and the results will be more reproducible.

## Concluding remarks

In summary, it is debatable whether the reduction and alkylation of proteins between the two dimensions of 2DE is necessary. Based on its widespread use in laboratories, the routine 2DE workflow still needs to be optimized regarding this step. We strongly suggest performing reduction and alkylation prior to IEF. Currently, protein sample preparation is usually conducted at reducing conditions in the presence of DTT; therefore, adding protein alkylation step to the sample preparation process will not increase the complexity or duration of the protocol. The application of reducing and alkylating reagents (working in concert in the extraction medium) or commercial kits should be optimized according to protein samples, especially the efficiency of protein alkylation during sample preparation. We also recommend that the additional equilibration step between the two dimensions is omitted if the reduction and alkylation of proteins has been incorporated in sample preparation, and that sample preparation protocols are optimized to ensure that the disulfide reduction is completed. This modification would reduce the potential loss of proteins and the “blurring” of focused spots that often occurs in 2DE gels.

## Author contributions

WW: Conceived the paper. WW, XW, and CX: Wrote the paper.

### Conflict of interest statement

The authors declare that the research was conducted in the absence of any commercial or financial relationships that could be construed as a potential conflict of interest.

## Abbreviations

2DE: two-dimensional gel electrophoresis
DIGE: difference gel electrophoresis
DTT: dithiothreitol
IAA: iodoacetamide
IEF: isoelectric focusing
IPG: immobilized pH gradients
2-ME: 2-mercaptoethanol
SDS-PAGE: second-dimension sodium dodecyl sulfate polyacrylamide gel electrophoresis.
